# Natural environments, nature relatedness and the ecological theater: connecting satellites and sequencing to shinrin-yoku

**DOI:** 10.1186/s40101-016-0083-9

**Published:** 2016-01-13

**Authors:** Jeffrey M. Craig, Alan C. Logan, Susan L. Prescott

**Affiliations:** 1 Murdoch Childrens Research Institute; Department of Paediatrics, University of Melbourne, The Royal Children’s Hospital, Flemington Road, Parkville, Victoria 3052 Australia; 2 CAMNR, 23679 Calabassas Road, Suite 542, Calabassas, CA 91302 USA; 3 School of Paediatrics and Child Health, Princess Margaret Hospital for Children, University of Western Australia, GPO Box D 184, Perth, WA 6840 Australia; 4 International Inflammation (in-FLAME) Network, Worldwide Universities Network (WUN), Perth, Australia

## Abstract

Recent advances in research concerning the public health value of natural environments have been remarkable. The growing interest in this topic (often housed under terms such as green and/or blue space) has been occurring in parallel with the microbiome revolution and an increased use of remote sensing technology in public health. In the context of biodiversity loss, rapid urbanization, and alarming rates of global non-communicable diseases (many associated with chronic, low-grade inflammation), discussions of natural vis-a-vis built environments are not merely fodder for intellectual curiosity. Here, we argue for increased interdisciplinary collaboration with the aim of better understanding the mechanisms—including aerobiological and epigenetic—that might help explain some of the noted positive health outcomes. It is our contention that some of these mechanisms are related to ecodiversity (i.e., the sum of biodiversity and geodiversity, including biotic and abiotic constituents). We also encourage researchers to more closely examine individual nature relatedness and how it might influence many outcomes that are at the interface of lifestyle habits and contact with ecodiversity.

## Viewpoint

The study of physiological anthropology concerns itself with understanding the ways in which the modern environment exerts selective pressures on humans and to what extent those pressures influence physiology. As such, physiological anthropology is ultimately a study of health and well-being and enjoys a special place as a central hub between countless branches of science and medicine. At times, some of the research developed through other disciplines—public health, psychology, environmental sciences, and ecology to name but a few—may seem disconnected from physiological anthropology. Conversely, some of the research produced by experts in physiological anthropology may not be immediately apparent to those in other isolated fields.

Here, in this invited Viewpoint, we will attempt to sew together some of the rapidly accumulating research on natural environments and identify ways in which biodiversity (and products of biodiversity) interact with psychological constructs (under the rubric of “nature relatedness” or an individual’s connection to nature). The aim of this effort is to highlight that there are many potential linkages between psychology and physiology in the modern environment that have yet to be married. As described below, an individual’s connection to nature is associated with health and well-being. We wonder, what are the physiological connections between the two? These have not been explored. Could it be that physiological changes in a certain environment impact on nature relatedness?

Moreover, a growing body of research utilizing satellite technology has demonstrated clear associations between natural environments and a wide variety of positive health outcomes. Yet, the interpretations of such research remain limited because mechanisms—including those pertaining to physiology and psychology—remain largely unexplored. Finally, a third area of research moving at a brisk pace is that pertaining to the microbiome. Thanks in large part to advances in high throughput sequencing and other methods, we can now study human and environmental bacterial communities with unprecedented precision. Again, we highlight that these advances are of great relevance to physiological anthropology because the microbiome research may also explain much about physiology in the modern environment. However, this too may be a product of individual relationships to the modern environment.

Here, we will examine some important history and use research advances (starting in the “Advances in research” section) to highlight that there are potentially fruitful lines of research that could link what is already known concerning in vivo research on natural environments (exemplified by Japanese *shinrin-yoku*), public health data gathered from satellites, microbes (and products of biodiversity), and individual connection to the environment—all with an eye toward expanding what is known on the physiological plane. We do so by acknowledging at the outset that some of the connections are speculative. Some have only limited research to support them. Yet, in a rapidly changing, urbanizing world, we suspect that the study of these potential interconnections is an urgent task not only for physiological anthropology but also for science and medicine.

## Forests and physiology

When the giant of the forest dies and falls to the ground, it is by the action of the bacteria that the tree trunk gradually disappears from view…this is the secret of nature’s eternal freshness…dissipated in the air or into simple compounds which sink into the Earth. Red Cross Notes on Useful Bacteria, 1900 [1]

Twenty-five years ago, Japanese scientists traveled to Yaku island (Yakushima; *shima* = island in Japanese) to determine if spending time in a forest environment might influence mood and markers of stress physiology. Although a preliminary study based on a very small sample, the results indicated that walking in a forest environment could provide a lift in mood and reduce objective markers of stress physiology [2]. The first small step toward proper in vivo investigation of natural environments was taken; Yakushima as a location to take that giant leap for humankind was highly appropriate.

Yakushima includes a ≈19,000-ha area designated as a biosphere reserve. Sitting at a biogeographic boundary between tropical and temperate regions, the island contains a remarkably rich variety of flora. Walking on this small island translates into *visual* encounters with evergreen broadleaf forests, conifer, 2000-year-old cedar trees, and nearly 2000 different plant species [3]. Pioneering researchers exploring the effects of the forest on mood and stress were not merely interested in the visual.

Throughout the 1990s, Japanese researchers started asking fundamental questions. Does walking in a forest influence mood state and stress physiology? And if so, might the forest environment specifically amplify the benefits of generalized physical activity? Could the forest environment influence stress physiology by olfactory or other non-visual pathways?

Researchers set their sights on phytoncides, a generalized term for natural chemicals released by plants into the environment. It was theorized that these chemicals could influence stress physiology through inhalation. Preliminary findings were published in an English-language textbook [2], and work on forest-based exercise therapy in patients with diabetes was reported in the *International Journal of Biometeorology* (IJB) in 1998—marking the first time that the Japanese term *shinrin-yoku* would be uploaded to PubMed [4].

The term *shinrin-yoku* struck a chord. More than a visual (esthetically pleasing) experience in nature, s*hinrin-yoku* was described as forest-air bathing, with an emphasis on what was encountered during *breathing*. The *shinrin-yoku* experience allowed an individual to literally take in the “components emitted from the forest” [4]. Since forest air is rich in volatile and non-volatile components and other unseen constituents that might be absent (or found in lower quantities) in urban built environments, *shinrin-yoku* was not merely an escape from toxic urban air [2, 4]. Walking through forests on Yakushima, therefore, was an opportunity to visualize, touch, listen to, and inhale nature. It meant *bathing* in biodiversity.

## Aerobiology and the hygiene hypothesis

At the same time and along somewhat related lines, experts in aerobiology were also beginning to turn their attention to the health implications of natural air constituents. This new research direction involved environmental biodiversity and the microbial constituents that may or may not be encountered by humans in the near-surface atmosphere of various settings. Writing in the textbook *Perspectives in Environment* (1998), researchers highlighted the health implications of airborne biodiversity in the context of allergic disease:

“In recent years, special attention has been given to the biodiversity of airborne biocomponents. These biocomponents are present in the air in the form of pollen grains, fungal spores, bacteria, mycelium, cysts, algal filaments and spores, lichens, insects and their parts, plants and animal tissues, and several other microorganisms. The presence of such biomatter in the air depends upon the occurrence of diversity of flora and fauna in the surroundings.” [5]

This interest was undoubtedly fueled, at least in part, by the so-called hygiene hypothesis. The generalized idea of the original hypothesis and its variants is that the global rise in allergic disease could be related to diminished opportunity for early-life exposure to pathogenic (and diverse commensal) microbe exposure via increased hygiene, antibiotic use, smaller family sizes and lower exposure to bacteria in foods, and the overall environment [6, 7]. The end result of altered microbial contact in modern environments was an “abnormally stable microflora” [7]. It has been theorized that this “new normal” creeping into developed, urbanized, industrial nations, a microbiota of modernity, might not be in the best interest of health promotion.

## Eye in the sky

As international researchers toiled away at identifying airborne biocomponents (still largely reliant upon culture technique for microbial evaluation), and while the first Japanese research subjects walked through forests, earth observation satellites were in orbit several hundred miles above them. Among other things, the satellites dedicated to earth science were measuring different wavelengths and intensities of visible and near-infrared light reflected by the land surface. This allowed for a normalized difference vegetation index (NDVI) of “greenness” or vegetative vigor [8].

In the late 1990s, the utility of satellite technology to assist in land-based ecological assessments—measures of biodiversity—was beginning to take shape [9]. However, the initial public health application of NDVI was understandably in priority order—assessments and modeling of drought, disease vectors, threats to food supply, environmental contamination, and so on. It would take a bit of time before remote sensing would break through as an important tool to examine broad health and well-being endpoints.

## Advances in research

The application of remote sensing as a means to link residential proximity to green natural environments and risk of chronic, non-communicable diseases, socioeconomic disparities, and other public health outcomes has witnessed tremendous growth. Layered upon large cohort data and the results in favor of a green space benefit are literally visible in the health outcome statistics [10–15]. Of course, the NDVI does not tell us whether or not a person actually *uses* natural environments for physical activity and social engagement. Furthermore, emerging studies are indicating that access to natural environments and public open spaces may be intertwined with complex non-communicable diseases (NCDs) determinants such as dietary patterns [16–20]; however, there are likely to be untold benefits of simply having good quality green space in the residential proximity [21]. The most obvious might be clean air [22].

Field research involving natural environments has also grown. While still requiring larger sample sizes and replication of existing work, studies involving *shinrin-yoku* (now generally referred to in Japanese studies as simply “forest medicine” or “forest therapy”) have grown in sophistication. Spending time in a forest environment has been linked with decreased cortisol, inflammatory cytokine (and chemokine) production, lowered blood pressure, improved heart rate variability, and elevations in natural killer cell activity [23–29].

These outcomes are highly relevant to the current epidemic of chronic, inflammatory NCDs. In addition, research designs now involve urban built environment (control) exposure, gender differences, personality features, seasonal influences on foliage, and experimentation with forest components such as sounds and airborne phytoncides [30–33]. The studies in natural environments have also enjoyed technological advances that allow for more convenient in-field assessments (e.g., electroencephalogram, cortisol).

Then we have the so-called microbiome revolution. Discussions of the possible use of beneficial microbes for NCD prevention and/or treatment, including mental health, are no longer met with blank stares and yawns. Advances in culture-independent, high-throughput sequencing technology have woken the disinterested from their slumber. Microbes matter. Although there are far more questions than answers, the relevancy of indoor and outdoor microbial contact to health has now moved closer to center stage [34–37].

In sum, the early origins of each section of primary research discussed thus far—in vivo experiments on natural environments, the use of remote satellite technology to access greenness in public health, and examination of airborne (including living constituents)—have grown leaps and bounds. Largely, they have developed apart from one another. Now, we will attempt to reconcile some of this research, or at least its possibilities, in ways that might benefit the stimulation of future research ideas.

## Microbes, phytoncides, ions—air to brain?

Microbes are a less obvious part of the biodiversity encountered on walks through natural environments. We know with a greater degree of certainty that the bark of trees, the stems, leaves, and needles of forested and vegetation-rich areas are teeming with microbial diversity [38–40]. Forest soils are also incredibly rich sources of microbial diversity [41]. We know that microbes, including those from the soils and leaf/needle surfaces of forests, are readily dispersed in the air [42–44], and we also know that Yakushima has some very interesting soil microbes [45]!

Microbes also interact with two other “unseen” constituents of the near-surface atmosphere in natural environments—phytoncides and air ions. Numerous studies have found a relatively higher concentration of negatively charged ions within natural environments, especially within forests and areas close to bodies of water [25, 46, 47]. Waves, whitecaps, and waterfalls can produce a relative increase in negative ions [48] and also promote the ejection of microbes into the atmosphere [49].

Research concerning negative ions has been clouded by marketing overreach associated with commercial ion-generating machines. However, evidence does point to beneficial effects on mood and inflammation [50, 51]. Phytoncides and negative ions enjoy a bi-directional relationship in natural environments, with higher concentrations of one influencing the other [52]. Of course, phytoncides are a product of plant interaction with microbes, and both phytoncides and negative ions may dictate the microbial makeup in the air within a natural environment. Dubos cites older research wherein the relatively high concentration of positive air ions was associated with decreased intestinal lactobacilli in infants [53]. Examining interactions between phytoncides, microbes (skin, intestinal, lung), and air ions with more sophisticated modern techniques is an area ripe for research in physiological anthropology.

In the meantime, it would seem obvious that microbes are part of a complex discussion concerning what might (or might not be) inhaled within differing environments. The field of microbiome research is sufficiently advanced to put forward a theoretical framework of how airborne microbiota—both directly and indirectly—can influence the human brain and behavior. Skin microbiota, no longer viewed as an “external” entity, are capable of influencing systemic immune function [54, 55]. Research shows marked differences in the skin microbiota or rural and urban residents [56]. The intra-nasal administration of non-harmful microbes can also influence systemic immune signaling [57–59].

The inhalation of phytoncide is known to reduce physiological stress [60], and how that might impact intestinal microbiota in the context of the emerging gut-brain-microbe research is unclear. Given the detrimental influence of stress on the microbiome [61], it would be easy to speculate that stress reduction could temper dysbiosis. Animal research involving the oral administration of very minor amounts of phytoncides derived from Korean pine (a mere 0.2 % of total dietary intake) has shown that phytoncide can improve gastrointestinal microbiota profile and nutrient digestibility [62].

Reviewed in detail elsewhere [63], pathways connecting intestinal microbes to brain include communication via the vagus nerve, immune-mediated pathways (including microglia [64]), intestinal barrier maintenance, limitation of oxidative stress, enhancement of nutrient bioavailability, and neurotransmitter precursors. Current evidence also suggests that changes to the microbiome results in widespread changes to gene expression in affected organs and in organs such as the brain. Such changes have been shown to be mediated by epigenetic factors which can lead to long-term changes in gene expression without a change in DNA sequence [65]. Epigenetic mechanisms involve the binding of small molecules to DNA and the histone proteins responsible for packaging and thereby regulating DNA. Such mechanisms include methylation of the CpG dinucleotide in DNA, acetylation and methylation of histones, and the binding to DNA of small non-coding RNAs.

Although diet has been the primary research focus concerning intestinal microbial diversity, we take this opportunity to highlight that other external environmental factors also seem to play a role in how intestinal microbial communities take their shape [66–70]. In other words, it is entirely possible that natural environments can impact upon all human-associated microbial communities, which in turn could influence nerve cell communication. Taken together, the above evidence provides plausible mechanisms by which external environmental (including airborne) microbiota can bring about feelings of pleasure and change our mental and physical health for the better.

## *Vis medicatrix naturae*

Although it has taken quite a few years, these seemingly disparate areas of research—forest walking, exploration of the unseen components of air within natural environments, and green space epidemiology via remote sensing—have begun to intersect. In their own ways, each area of research has been evaluating the extent to which natural environments might work toward health *promotion* [71, 72]. The available evidence suggests that natural environments might influence mood and stress physiology. A buffering of allostatic load—reduction of the destructive tandem of low-grade chronic inflammation and oxidative stress—can provide a potential mechanistic link to both healthy birth outcomes and mortality reduction related to NCDs [73].

We began our viewpoint discussions with Yakushima not simply because it was the birthplace of the *scientific* investigation of *shinrin-yoku*. Islands are hotbeds of plant diversity—20 % of the world’s plant species are found only on islands. Yakushima, with its various climate zones and rich geological history, represents the need to bring the natural environments-public health research trail toward the question of diversity.

There are hints that the value of natural environments in relation to complex non-communicable diseases (mental health in particular) may be a by-product of biodiversity [74–79]. What sort of diversity is the question? It could be diversity in species of trees and/or vegetation and birds (and the audible sounds of diversity). It might be encounters with non-harmful environmental microbes (especially those encountered in less urbanized, traditional societies) and/or the natural airborne chemicals that are a product of biodiversity (e.g., phytoncides) secreted from trees and other plants [80, 81], or it could be a synergistic combination of the lot?

Of course, natural environments are so much more than their biotic components. A century ago, biologist Sir John Arthur Thompson proposed that human evolutionary connections to natural environments were being eroded by urbanization. In his keynote address to physicians of the British Medical Association, he focused on *vis medicatrix naturae* (the healing power of nature) and interpreted it to be clinically relevant, mindful contact with the biotic and abiotic elements of nature. As he said: “We have put ourselves beyond a very potent vis medicatrix if we cease to be able to wonder at the grandeur of the star-strewn sky, the mystery of the mountains, the sea eternally new…” [82].

## Philia overload: ecodiversity and human health

“The study of plants and animals carries an impressive lesson as to the unity prevailing amid all the diversity of Nature…the grand outcome of geological study is that it brings vividly before the mind the immensity of time…the contemplation of past geological ages, reckoned by millions of years, the fact that our Earth is coeval with the sun in age - all these considerations tend to immeasurably expand our mental horizon, and thus to react in a way to broaden the mind.” Alpheus S. Packard, Brown University, 1892 [83]

Although biologist Edward O. Wilson did not coin the term biophilia (see [71] for its historical use), in 1979, he argued that it is “the rich, natural pleasure that comes from being surrounded by living organisms, not just other human beings but a diversity of plants and animals” [84]. Almost two decades earlier, geographer Yi-Fu Tuan introduced the term topophila, originally defining it as a “love of nature” [85] and subsequently as “the affective bond between people and place or setting” [86]. Landscape architect Robert Thayer suggested that geophilia or gaiaphilia might be more appropriate terms for a generalized love of the land and the Earth, including its abiotic parts [87].

It is not our desire to introduce yet another *philia* into the natural environments discussion. However, it is becoming increasingly clear that geodiversity—the variability of the Earth’s surface materials, landforms (e.g., mountains, bodies of water) and physical processes—is highly relevant to biodiversity, ecosystem services, and conservation [88, 89]. It is interesting to note that in human research directed at the evaluation of the primary domains underlying Tuan’s topophilia, only the ecodiversity category emerged as significantly and positively correlated to respondents’ quality of life [90].

Areas with noted plant species diversity, including forests, are often at the rich end of geodiversity. They are typically mountainous and sitting on a steep climate gradient (Yakushima has both of these attributes) or areas such as Southwestern Australia, California, and other world regions that enjoy a Mediterranean climate [91]. Throughout history, our genus has been evolving at the genetic and epigenetic level because living organisms, as John Arthur Thompson stated, “enter into subtle inter-relations with their inanimate environment, whence also new complexities spring” [92]. Geodiversity is not only part of those complexities; in the context of ecosystem services, it is also a matter of human health and well-being.

In 1996, the biotic and abiotic environmental terms were incorporated into a simple concept of ecodiversity. As defined by Barthlott, ecodiversity is the total diversity of a region, including the sum of its biodiversity and geodiversity [93]. It also considers the highly relevant factor of climate. Natural environments and human health, particularly mental health, are obviously discussions involving natural light, temperature, and weather [94, 95]. Weather-related factors may also determine the microbiota with which one may make contact [96]. As such, ecodiversity underscores that biodiversity is dependent upon a host of abiotic factors—considerations of which pass through the expertise of many scientific disciplines.

If we truly seek to encourage interdisciplinary dialogue and liberate silo-sequestered research related to public and planetary health, then perhaps adoption of the term ecodiversity is not merely name-game semantics. The biophilia hypothesis posits that humans have an innate need for affiliation with other forms of life, most notably the visible portions of life such as savannah-like trees. It was an extension of JA Thompson’s 1914 *vis medicatrix naturae* hypothesis. Rene Dubos continued the theme of “universal fellowship with all other human beings and even with other forms of life” [97] in his *biological joie de vivre* hypothesis described elsewhere [71].

As a microbiologist, Dubos also forewarned (nearly five decades ago) of the NCD epidemic that might precipitate via a disconnection from the microbes with which we co-evolved. The recent biodiversity hypothesis further proposes that loss of biodiversity at the level of macrobiota will translate into loss of contact with microbiotic diversity. Specifically, “biodiversity loss leads to reduced interaction between environmental and human microbiotas. This in turn may lead to immune dysfunction and impaired tolerance mechanisms in humans” [98].

If only for convenience, it might be helpful to roll these and other overlapping hypotheses related to modern lifestyle mismatches into a universal evolutionary health concept that considers contextual biotic and the abiotic determinants of the so-called diseases of civilization. Famed ecologist G. Evelyn Hutchinson folded the abiotic/biotic into the metaphor of an “ecological theater” in which an “evolutionary play” is taking place [99]. For the better part of three million years, our genus has been a player on that stage, in many ways like actors in a long-running Broadway play. The stage and the production of the play have, of course, changed to some degree over space and time. However, modern human lifestyles have rapidly changed both the theater and the play.

Diminished contact with evolutionary-rooted ecodiversity appears to be a product of modernity. This can be extended to inclusion of those aspects of the modern landscape and lifestyle that are incompatible with levels of adequate natural daylight and very low levels of light at night under which humans evolved. However, at this point, lack of contact with ecodiversity is not well established in its linkage to NCDs vs. other lifestyle contributors, such as higher consumption of ultra-processed foods and sedentary behavior. The role of urbanization on biodiversity is complex and may, in certain cases, actually increase species richness at the cost of native species [100].

At present, there is little evidence to show that the promotion of human health, reduction of NCD risk, and the minimization of psychological distress are sitting along a neatly defined and universal continuum in favor of the countryside and biodiversity [101, 102]. The role of biodiversity loss in elevated *communicable* disease risk is becoming more clear [103]. As yet, early life experience with microbial exposure via traditional lifestyles has not been proven to differentially alter later-life immune responses in complex mental disorders such as depression [104]. Moreover, urbanization does not appear to automatically translate into significant differences in dominant microbial communities in the outdoor air, at least not in North America [105].

## Many unanswered questions

Despite recent advances, and much needed convergence of research pathways, there are many unanswered questions concerning natural environments and their utility in policy and practice. Basic questions are now obvious, including those related to “dose”. For example, how often and for how long does one have to visit a natural environment in order to enjoy meaningful health-related benefits? Does the type of natural environment—its physical features, spatial arrangement, biodiversity, geodiversity, climate, weather, density of trees, sounds, smells, microbiota, natural light, air ions, soils, and sand—matter to health outcomes?

It also seems reasonable to ask if time of day matters, or if an optimal time to spend time in natural environments might exist? We would speculate that such a time might be early morning. There are several features of natural environments in the morning that could differentiate mood and/or physiological outcomes vs. afternoon. First, the mood-regulating [106] blue portion of the natural light spectrum is highest in the morning [107, 108]. Second, phytoncides are more abundant in the morning [109]. Third, culture-based techniques show that certain airborne microbes in forests and other natural environments (coastal, rural) are also significantly higher before noon [43]. And fourth, negative ions may also be relatively higher in the morning [110, 111].

There are also required discussions concerning the dangers that might exist in natural environments—including but not limited to disease-carrying vectors, predators, allergens, and excess exposure to ultraviolet radiation. We need to learn more concerning the physical aspects of natural environments that might actually induce fear and compromise the endgame of outcomes such as stress reduction and improved vitality [112]. Not every study has found natural environments to be associated with positive health outcomes [113, 114], and these “negative” studies may provide essential information concerning the limitations of green space relative to other biopsychosocial factors.

## Connection to nature

It seems evident that not every person who lives close to natural environments, or walks through a lush forest, is going to receive the same benefits. To what extent are previous experience with and perceptions of nature guiding those differences? Nature relatedness (NR) (and similar psychological constructs such as nature connectivity, nature connectedness) have been connected to mental well-being [115, 116]. Briefly, the NR and other such scales can measure an individual’s fascination with, interest in, and desire for nature contact. Moreover, the measurements go beyond superficial “love of nature”; they can also tap into awareness and understanding of the natural world, including the bits of ecodiversity that might not be aesthetically pleasing.

We would encourage researchers to incorporate validated nature connectivity scales into research protocols. For convenience only, we point toward the validated six-question short form (nature relatedness-6; NR-6) that can rapidly capture an individual’s orientation to nature [117]. The NR-6 (and other scales) could be folded into a variety of studies that could provide critical unifying information; these findings could link many aspects of seemingly disconnected research. Here, we provide ten examples of what we consider to be worthwhile questions:

1. Is NR connected to healthier lifestyle habits? Dietary patterns? Fermented food consumption? Physical activity?
2. Is NR associated with differing skin, oral, or intestinal microbiota/microbiome?
3. Is NR associated with risk of NCDs? Is it connected to physiological markers of allostatic load? Could subgroups in epidemiological studies be evaluated for NR? Given the emerging studies on green space and healthy birth outcomes [118] and inverse associations between surrounding greenness and incident asthma development [119], we wonder how maternal NR might mediate these findings?
4. Is NR associated with prescription drug use, including psychotropics and antibiotics?
5. Is NR linked to genetics and/or epigenetics?
6. Is NR associated with screen time or time spent indoors? Is it connected to vitamin D levels?
7. Is NR associated with neighborhood features, perceptions of pollutants, and/or local biodiversity? Does NR place a higher burden on stress physiology while encountering human generated noise? For example, residential traffic noise is associated with increased risk of depression [120], and we wonder if those scoring high on NR may experience traffic noise as more of an irritant?
8. Is NR associated with disgust sensitivity, fear of microbes, aspects of hygiene?
9. Is NR related to brain activity while viewing nature vs. urban scenes?
10. Does NR influence outcomes of intervention studies involving prescription medications and/or eco-psychotropic agents such as aromatic chemicals (phytoncides), beneficial microbes, etc.?

Answers to these and other questions could provide an entirely new direction to research endeavors. If NR is so valuable to well-being—and numerous studies indicate that it is—then how do we cultivate it? Application of the NR scale in studies involving twins would provide tremendous insight. Thus, as far as learning more about the ways in which natural environments can influence health, the NR-6 could provide a research conduit between unseen satellites in space and invisible microbes on Yakushima island.

Finally, and by no means least important, we should strive to further our understanding of the ways in which natural environments might foster human interconnectedness. As mentioned earlier, natural environments are often places where the development of social capital can be fostered [121]. Community gardens might represent a prime example of this phenomenon.

However, it has been shown that NR is associated with empathy, and images of natural environments (as well as the presence of plants) can foster pro-social actions, including resource sharing [122–124]. Moreover, when researchers induce awe via nature scenes, subjects are more likely to report increased oneness to other human beings and a more pronounced willingness to do volunteer activities [125, 126]. Perhaps ecodiversity may play a role in connecting humans in as yet untold ways. Vital to the study of physiological anthropology, we can make attempts to understand how social gains may be manifested in physiological endpoints.

## Call to action

Remote sensing and land-based ecological assessments, along with more detailed examinations of just what, precisely, someone is breathing in while spending time in a forest or other natural environment [127], can be layered into experimental outcomes. We can further examine if the inherent value of physical activity and social engagement is amplified by environments rich in ecodiversity. Of course, such work can also help to strike the appropriate balance between health-promoting access to nature and essential conservation needs [128, 129].

Twenty-five years on, interdisciplinary collaboration can help bring the discipline of forest medicine (and more broadly, the therapeutic potential of natural environments) into a more prominent place within discussions of preventive medicine and public health. Much has changed in the last quarter century, especially concerning the tandem juggernaut of chronic inflammatory NCDs and climate change. Their collective force is already bearing down upon us [130–132], and as such, research exploring natural and built environments from a psychological and physiological perspective is not merely a matter of intellectual fancy. Personal, public, and planetary health is on the line.
